# Mosaic analysis of stem cell function and wound healing in the mouse corneal epithelium

**DOI:** 10.1186/1471-213X-9-4

**Published:** 2009-01-07

**Authors:** Richard L Mort, Thaya Ramaesh, Dirk A Kleinjan, Steven D Morley, John D West

**Affiliations:** 1 Division of Reproductive and Developmental Sciences, Genes and Development Group, The University of Edinburgh, Hugh Robson Building, George Square, Edinburgh EH8 9XD, UK; 2 MRC Human Genetics Unit, Crewe Road, Edinburgh, EH4 2XU, UK; 3 Clinical Biochemistry Section, Division of Reproductive & Developmental Sciences, University of Edinburgh, Centre for Reproductive Biology, Queen's Medical Research Institute, 47 Little France Crescent, Edinburgh EH16 4TJ, UK

## Abstract

**Background:**

The mouse corneal epithelium is a continuously renewing 5–6 cell thick protective layer covering the corneal surface, which regenerates rapidly when injured. It is maintained by peripherally located limbal stem cells (LSCs) that produce transient amplifying cells (TACs) which proliferate, migrate centripetally, differentiate and are eventually shed from the epithelial surface. LSC activity is required both for normal tissue maintenance and wound healing. Mosaic analysis can provide insights into LSC function, cell movement and cell mixing during tissue maintenance and repair. The present study investigates cell streaming during corneal maintenance and repair and changes in LSC function with age.

**Results:**

The initial pattern of corneal epithelial patches in *XLacZ*^+/- ^X-inactivation mosaics was replaced after birth by radial stripes, indicating activation of LSCs. Stripe patterns (clockwise, anticlockwise or midline) were independent between paired eyes. Wound healing in organ culture was analysed by mosaic analysis of *XLacZ*^+/- ^eyes or time-lapse imaging of GFP mosaics. Both central and peripheral wounds healed clonally, with cells moving in from all around the wound circumference without significant cell mixing, to reconstitute striping patterns. Mosaic analysis revealed that wounds can heal asymmetrically. Healing of peripheral wounds produced stripe patterns that mimicked some aberrant striping patterns observed in unwounded corneas. Quantitative analysis provided no evidence for an uneven distribution of LSC clones but showed that corrected corneal epithelial stripe numbers declined with age (implying declining LSC function) but stabilised after 39 weeks.

**Conclusion:**

Striping patterns, produced by centripetal movement, are defined independently and stochastically in individual eyes. Little cell mixing occurs during the initial phase of wound healing and the direction of cell movement is determined by the position of the wound and not by population pressure from the limbus. LSC function declines with age and this may reflect reduced LSCs numbers, more quiescent LSCs or a reduced ability of older stem cells to maintain tissue homeostasis. The later plateau of LSC function might indicate the minimum LSC function that is sufficient for corneal epithelial maintenance. Quantitative and temporal mosaic analyses provide new possibilities for studying stem cell function, tissue maintenance and repair.

## Background

Female mice, hemizygous for the H253 X-linked *nLacZ *transgene (here termed *XLacZ*^+/-^), are X-inactivation mosaics and show variegated patterns of β-galactosidase (β-gal) reporter expression in all of their tissues [1]. These mosaics have been widely used to study lineage relationships during development [2,3] but they can also be used to analyse maintenance of adult tissues by stem cells [4,5].

### Maintenance of the corneal epithelium by stem cells

The corneal epithelium is an excellent model system for the study of tissue maintenance and repair because it is a discrete 5–6 cell thick epithelium replenished by a regionalised stem cell population, which is confined to the basal layer of the limbus at the periphery of the cornea [6,7]. These limbal stem cells (LSCs) produce transient (or transit) amplifying cells (TACs), which proliferate rapidly and migrate centripetally in the basal epithelial layer until their final division when both daughter cells move into the superficial layers, differentiate and are eventually lost from the epithelial surface by desquamation [8-11]. Previous studies with *XLacZ*^+/- ^mosaics from our group have shown that LSCs become active after birth [4] and identified possible genetic influences on LSC function [5].

**Figure 1:**
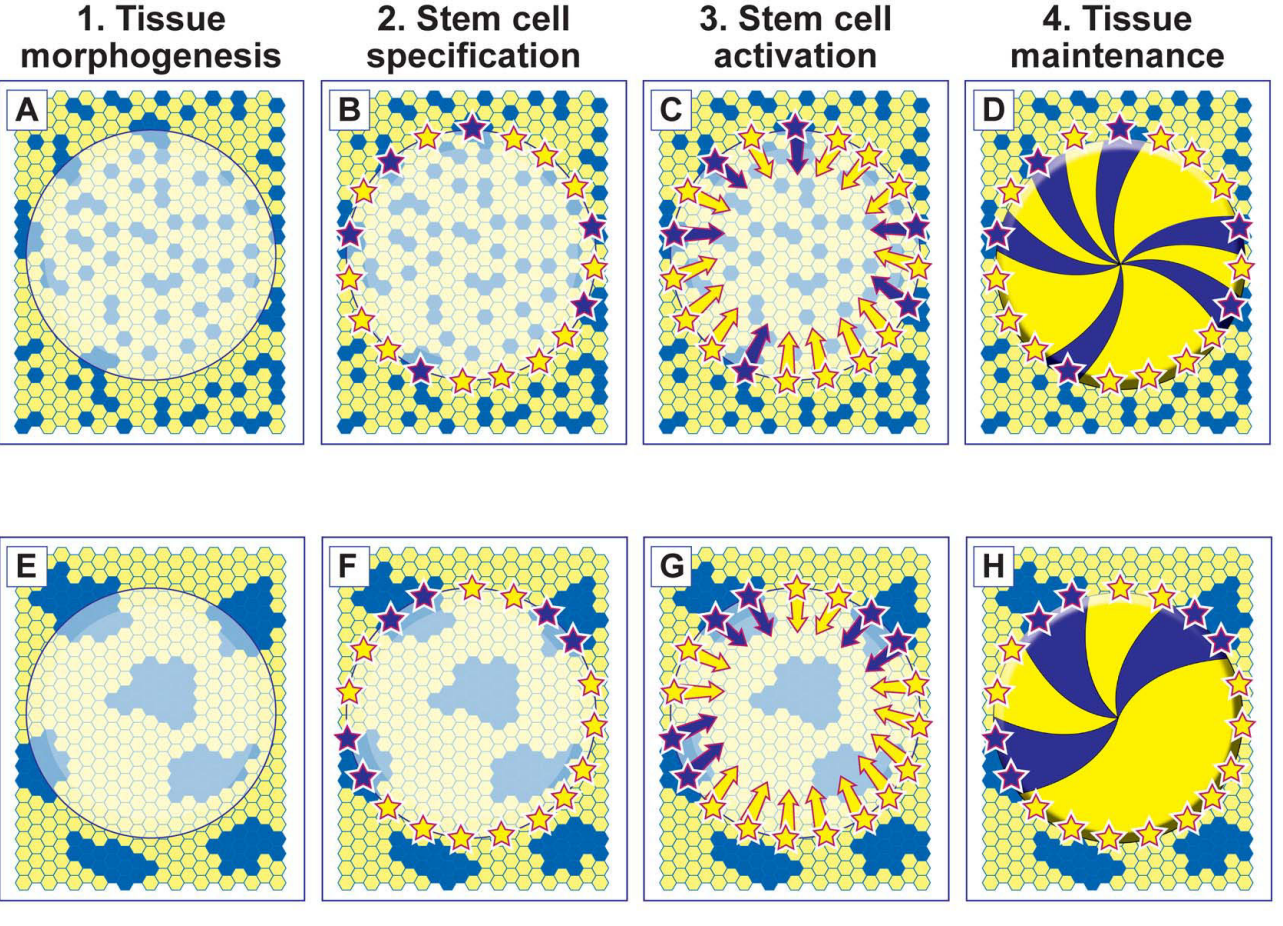
**The effect of cell mixing during development on the number of corneal epithelial stripes in an adult mosaic eye**. The following sequence of events is proposed to occur in a mosaic eye comprising a mixture of blue (β-gal-positive) and yellow (β-gal-negative) cells. 1. During tissue morphogenesis the regions designated to become the corneal, limbal and conjunctival epithelia are determined. (The surface ectoderm is shown as a mosaic patchwork of pale blue and yellow cells. The translucent disk represents the cornea with the limbus at its perimeter.) 2. Some of the basal limbal epithelial cells are specified as LSCs (shown as blue and yellow stars). 3. Some (perhaps all) of the LSCs become activated and produce TACs that migrate centripetally (blue and yellow arrows). 4. The tissue is maintained by centripetal migration of LSC progeny forming striping patterns. Two alternative outcomes are highlighted in A-D and E-H. A-D: The two cell types (blue and yellow) mix extensively (non-coherent clonal growth). Therefore, each LSC is likely to be specified from a different coherent clone even where adjacent LSCs are the same colour (e.g. neighbouring yellow stars). E-H: If there is less mixing during morphogenesis (limited coherent clonal growth) some adjacent LSCs will probably arise from the same coherent clone of surface ectoderm cells and so some LSC coherent clones will comprise more than one specified LSC. In E-H the corrected stripe number will under-estimate the number of active LSCs.

Our previous mosaic analysis, suggesting that LSCs become active after birth, was based on a transition from a pattern of patches to one of radial stripes. The sequence of events shown in Fig. 1A–D proposes that some of the basal limbal epithelial cells are specified as LSCs after the limbal epithelium has been determined. Subsequently LSCs become activated and the cornea is maintained by centripetal migration of TACs. Thus, it is predicted that the initial mosaic pattern of patches is established during fetal and early postnatal development and the emergence of stripes indicates when stem cell function begins. Stem cells are required to maintain tissues throughout life and the idea that stem cell function may decline with age and so contribute to age-related changes in tissue homeostasis is currently of great interest [12-14]. However, this possibility has not yet been investigated systematically for LSCs maintaining the corneal epithelium.

### Wound healing in the corneal epithelium

LSCs are involved in wound healing, as well as normal tissue homeostasis, and they are up-regulated to replace the lost cells [15]. Corneal epithelial wound healing proceeds through three stages: (i) an initial migratory stage to cover the wound with a cell monolayer, (ii) a proliferative stage to restore the epithelial thickness and (iii) a period of differentiation to restore the complex epithelial structure [16]. Several possible mechanisms could drive cell movement during the initial migratory stage, including population pressure of streams of cells moving centripetally from the limbus, population pressure from the wound-margin or other forces, such as chemotaxis or electric fields [17]. Cell proliferation is stimulated in the peripheral limbal epithelium and to a lesser extent in the corneal epithelium, within 12 hours of wounding, to replace lost cells [6]. However, proliferation at the wound margin may be suppressed to maintain tissue integrity [18]. Thus, many new cells will arise in the periphery and migrate centripetally, as in normal tissue homeostasis, to restore cell numbers and tissue morphology.

The source of the cells and the extent of cell mixing during the early phase of wound healing can be investigated experimentally by a combination of mosaic analysis and organ culture. Wound healing during 24-hour organ cultures reproduces the initial rapid movement of surrounding corneal epithelial cells to cover the exposed stroma but after 24-hours the experimental wound is only covered by a single layer of cells and the epithelium does not stratify during this initial *ex vivo *healing response [19-21].

The alternative experimental approach of wounding cultured corneal epithelial cell monolayers is thought to cause a loss of spatial constraints and induce motility of sheets of epithelial cells rather than individual cells [22]. However, this may not reflect the situation *in vivo *or in organ culture where wounding of a multi-layered stratified epithelium allows more scope for cell mixing during wound healing. This is because cells from the upper epithelial layers could contribute to the cell monolayer that forms during the initial movement phase. It has also been suggested that cells at the wound margin become less adhesive and may detach from the epithelial sheet [16], so promoting cell mixing. Distributions of retrovirus-labelled skin epithelial cells during *ex-vivo *wound healing [23] have been interpreted to suggest cell mixing is quite extensive [24] but it has yet to be determined to what extent cell mixing also occurs during corneal epithelial wound healing.

### Quantitative mosaic analysis of limbal stem cell function

Quantitative analysis of distributions of the two cell populations in mosaic tissues (e.g. analysis of the relative numbers, size, shape and distribution of patches) can provide more information than qualitative mosaic analysis [25-27] but this has not yet been widely exploited. The striping patterns in the adult cornea are produced by LSC function and cell movement in the epithelium. LSC function can be compared in different experimental groups by quantitative analysis of stripe numbers.

In an adult corneal epithelium, showing mosaic expression of a *LacZ *transgene, the stripes of β-gal-positive cells are elongated patches formed from one or more β-gal-positive coherent clones whose descendents have migrated centripetally. (The terms 'patch' and 'coherent clone' are defined in the Methods section.) A stripe spans the corneal radius, so its length is not affected by the number of LSCs and is not relevant to the analysis. The stripe width, however, is variable and depends in part on the number of adjacent corneal epithelial coherent clones belonging to the same cell population (either β-gal-positive or β-gal-negative). Clearly, an individual stripe is more likely to be made up of multiple adjacent β-gal-positive corneal epithelial coherent clones when the proportion of β-gal-positive cells in the corneal epithelium is higher. This source of variation in stripe width can be factored out by dividing the observed mean width of β-gal-positive stripes by the function 1/(1-*p*), where *p *is the proportion of β-gal-positive cells around the circumference [25,28]. The resultant 'corrected mean stripe width' can be used to derive a 'corrected stripe number' (see Methods section). This is proportional to the number of corneal epithelial coherent clones and can, therefore, be used to compare LSC function in different groups. A coherent clone of β-gal-positive limbal stem cells will produce a coherent clone of cells in the basal layer of the corneal epithelium that extends to the centre as cells move centripetally and extends to the suprabasal and outer epithelial layers as cells leave the basal layer. Each corneal epithelial coherent clone is assumed to be formed from a single active coherent clone of LSCs. Thus, the number of active LSC coherent clones can be compared in different groups of mosaic eyes by comparing the corrected stripe numbers.

Although, the corrected stripe number is related to the number of active LSC coherent clones it does not provide a direct estimate of LSC numbers. This is partly because the proportion of LSCs that are active may vary and also because the number of LSCs per LSC coherent clone may vary. For example, variation in the number of LSCs per LSC coherent clone may occur because of differences in the extent of cell mixing during development of the surface ectoderm, from which the corneal and limbal epithelia develop (compare Fig. 1A–D, showing the consequences of extensive cell mixing during development, with Fig. 1E–H, showing the consequences of less cell mixing).

### Aims

The aims of this study were to better characterise LSC function and the streaming and mixing behaviour of the cells they produce during maintenance, repair and ageing of the mouse corneal epithelium. Analysis of mosaic patterns in intact and wounded corneas demonstrated that (i) LSC function declines with age, (ii) little cell mixing occurs either during normal maintenance of the corneal epithelium or during wound healing, (iii) the main driving force during wound closure is not population pressure from centripetally streaming cells produced by LSCs and (iv) quantitative and temporal mosaic analyses provide new possibilities for studying stem cell function in tissue maintenance and repair.

## Results

### Qualitative characterisation of X-inactivation mosaic patterns in the corneal epithelium

**Figure 2:**
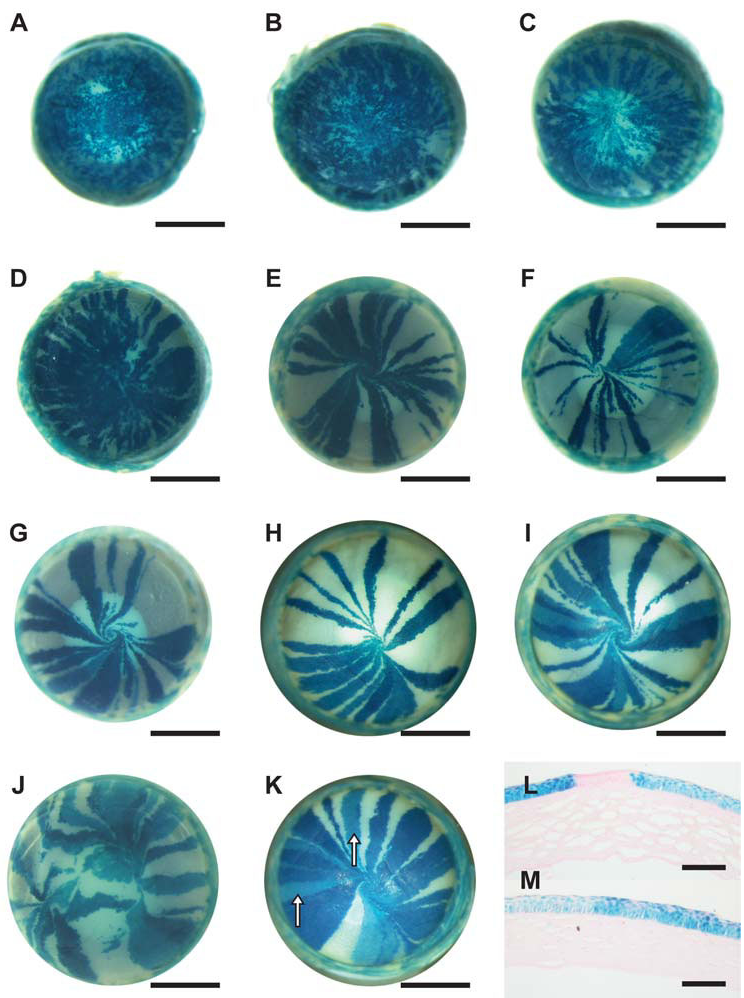
**Representative striping patterns in corneal epithelia of *XLacZ*^+/- ^mosaic mice**. Intact eyes from 3–52-week old *XLacZ*^+/- ^mice were stained for β-gal reporter activity. A: 3-weeks; B: 6-weeks; C: 8-weeks; D: 10-weeks; E: 15-weeks; F: 20-weeks; G: 26-weeks with an anticlockwise whorl; H: 39-weeks exhibiting a midline; I: 52-weeks with a clockwise whorl. J: Disrupted striping patterns in a wild-type eye appear to form a double whorling pattern. K: Some β-gal-positive stripes appear paler than others (white arrows). L: Section through a light blue and a dark blue stripe either side of a white stripe. M: Section through adjacent light and dark blue stripes. Scale bars in A-K = 1 mm. Scale bars in L-M = 100 μm.

**Figure 3:**
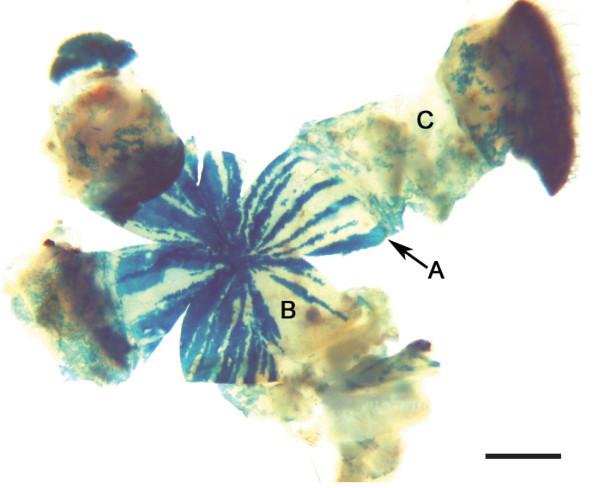
**The conjunctiva is not maintained by LSCs**. Whole-mount image showing the ocular surface of a female X-inactivation mosaic mouse. Stripes extend inwards from the edge of the limbus (A) to the corneal epithelium (B) and meet at the centre. There is no striping in any region of the conjunctival epithelium but mosaicism occurs as irregular-shaped patches (C).

Examination of mosaic corneal epithelia of 3–52 week old female *XLacZ*^+/- ^mosaic mice showed that the initial pattern of randomly orientated patches seen at 3 weeks was replaced by radial stripes by postnatal week 10 (Fig. 2A–D). This radial striped pattern was also present at all subsequent adult stages examined up to and including postnatal week 52 (Fig. 2E–I). The transition from patches to stripes was gradual and not completed until after 10 weeks (Fig. 2D,E), which is consistent with our earlier study [4]. This presumably reflects activation of LSCs between 3 and 5 weeks and the time required for subsequent replacement of the original mosaic patchwork pattern in both the basal and overlying suprabasal layers. The mature striping patterns (Fig. 2E–K) did not extend outwards into the conjunctival epithelium (Fig. 3), supporting earlier evidence that the conjunctiva is not maintained by LSCs [4,29].

Occasional abnormal mosaic patterns (Fig. 2J) may have been produced by wound healing (discussed below). Some β-gal-positive stripes were paler than normal (arrows in Fig. 2K) and histological sections showed that these pale stripes were composed entirely of pale blue cells in all of the epithelial cell layers (Fig. 2L–M), implying that pale stripes were caused by reduced β-gal expression rather than overlapping, misaligned β-gal-positive and β-gal-negative layers. This suggests that the source of the variation is clonal, presumably reflecting an epigenetic change in the level of β-gal expression in LSCs.

**Figure 4:**
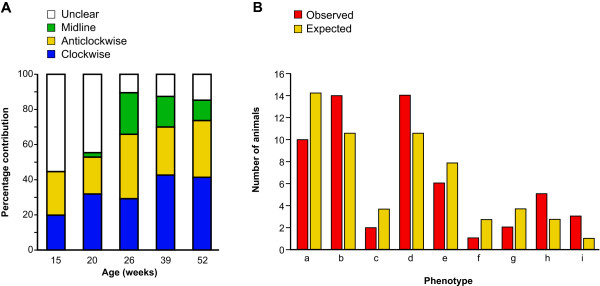
**Analysis of stripe patterns in the corneal epithelium**. A. Qualitative classification of stripe patterns at different ages. Eyes were classed as having a clockwise (n = 61) or anticlockwise whorl (n = 53), a midline (n = 21) or no clear pattern (n = 51). There was no change in the proportions of clockwise and anticlockwise patterns with age (χ^2 ^test; *P *> 0.05). B: Comparison of observed and expected frequencies of whorl phenotypes (a-i) in left and right eyes of 56 mice. Animals with unclear patterns in either eye were excluded. There was no significant difference between observed and expected frequencies (χ^2 ^test; *P *> 0.05) implying that there was no association between the phenotypes of the left and right eyes. Phenotype combinations: (a), clockwise (CW) left (L) plus CW right (R); (b), CW-L plus anticlockwise (ACW) R; (c), CW-L plus midline (ML) R; (d), ACW-L plus CW-R; (e), ACW-L plus ACW-R; (f), ACW-L plus ML-R; (g), ML-L plus CW-R; (h), ML-L plus ACW-R; (i), ML-L plus ML-R. The expected numbers of mice for each phenotype pair were calculated by multiplying the product of the two appropriate individual overall frequencies (CW = 0.455, ACW = 0.402 and ML = 0.143) by the number of pairs of eyes. For example, the expected number of mice with one CW and one ACW whorl is 0.455 × 0.402 × 56 = 10.25.

In adult mosaic corneas, the stripes often met at a central whorl (either clockwise or anticlockwise; Fig. 2G,I) or (less frequently) met at a midline to form a more bilaterally symmetrical pattern (Fig. 2H). Excluding eyes in which the pattern was unclear, 45.2% of 135 eyes from mice aged 15–52 weeks had clockwise whorls, 39.3% had anticlockwise whorls, and 15.6% had midline patterns (Fig. 4A). The proportion of unclear patterns decreased from 50% (37/74) at 15–20 weeks to 12.5% (14/112) at 26–52 weeks, implying that midline, clockwise and anti-clockwise patterns become more clearly delineated and easier to resolve after 20 weeks. The relative frequencies of clockwise and anticlockwise whorls were unaffected by age (Fig. 4A). For 56 pairs of eyes with clear patterns, the clockwise (51 eyes) anti-clockwise (45) and midline (16) patterns occurred independently in left and right eyes (Fig. 4B).

### Wounds in mosaic corneal epithelia heal clonally

**Figure 5:**
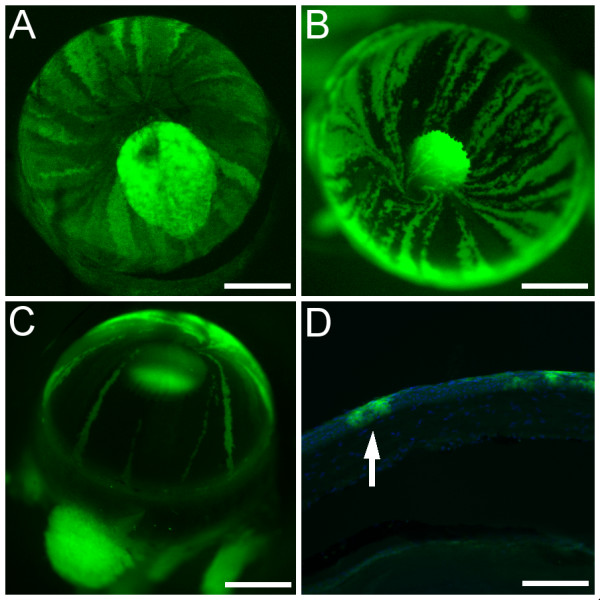
**Striping patterns in corneal epithelia of mosaic *PAX6-GFP *transgenic mice**. Fluorescence microscopy of corneas of *PAX6-GFP *transgenic mice from lines Y223 and Y001deltaDRR (described in the Methods section) showing green fluorescence of GFP-positive tissues. A: An eye from line Y223 with bright fluorescence from the lens. B,C: Eyes from 12-month old mice from line Y001deltaDRR showing radial stripes and whorls in the cornea. The bright green fluorescence seen in the pupil in A-C is GFP fluorescence from the lens. In A it also obscures part of the pattern of stripes in the cornea whilst in B and C it is reduced due to deletion of the DRR region of the *PAX6 *locus. D: Histological section showing DAPI-stained nuclei (blue) and both GFP-positive (green) and GFP-negative regions in the corneal epithelium. Arrow in D indicates a GFP-positive stripe, in which GFP-positive cells are aligned vertically in the epithelium. Scale bars: A-C, 1 mm; D, 100 μm.

In addition to *XLacZ*^+/- ^mosaics, the suitability of transgenic mice from lines Y001deltaDRR and Y223 [30] (both displaying mosaic corneal GFP expression) were evaluated for wound healing studies. These animals carry a yeast artificial chromosome (YAC) containing the human *PAX6 *locus [31] into which a GFP reporter gene has been inserted at the *PAX6 *ATG start codon, placing it under the control of the *PAX6 *regulatory elements. This also eliminates production of PAX6 protein from the YAC ensuring that wild type Pax6 levels in these mice are unaffected. Both lines demonstrated patterns of GFP-positive and GFP-negative radial corneal stripes (Fig. 5) qualitatively similar to those observed in *XLacZ*^+/- ^mosaics (Fig. 2). The reason for the mosaic transgene expression is not clear but it probably involves stochastic transgene inactivation early in development so that only a proportion of adult limbal stem cells express GFP. Line Y001 contains a single copy of the YAC with a 10–20 kb truncation, while Line Y223 contains six copies and in both cases tissue specific GFP expression patterns correspond to the endogenous pattern of Pax6 expression [30]. Line Y001deltaDRR was produced by crossing Y001 with the *CAGGS-Cre *line [32] resulting in germline deletion of the downstream regulatory region (DRR) and leading to severely reduced GFP expression in the lens compared to line Y223 [30]. Therefore, following initial evaluation, line Y001deltaDRR (*PAX6-GFP*) was used for the wound healing study because corneal stripes were clearer in eyes with weaker GFP expression in the lens. Histological sections showed that the GFP-positive stripes were localised to the corneal epithelium not the stroma (Fig. 5D) and were similar to the β-gal-positive stripes in the *XLacZ*^+/- ^mosaics (Fig. 2).

**Figure 6:**
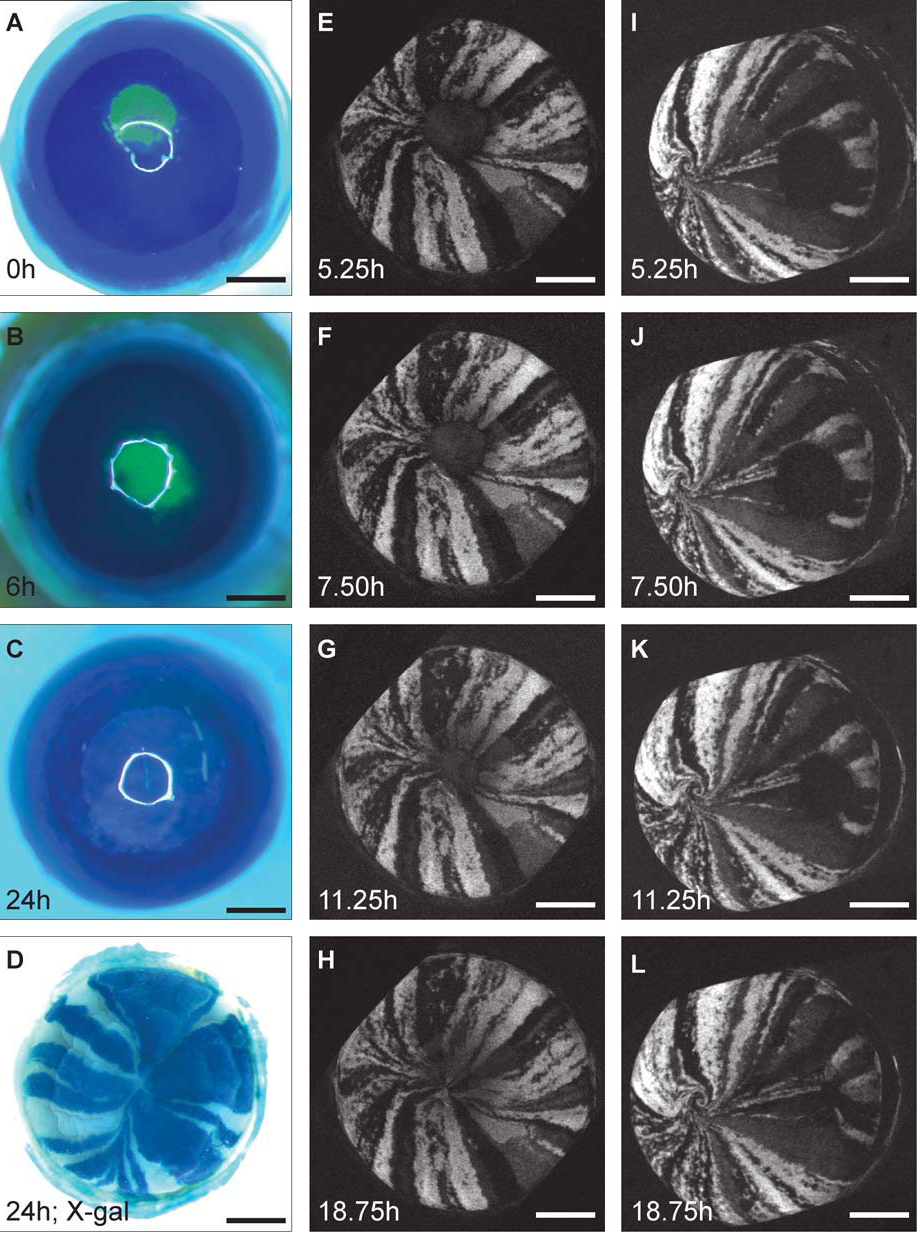
**Wound healing in mosaic mouse corneal epithelium restores the striping pattern**. A-C: Fluorescein staining of wound healing of *XLacZ*^+/- ^corneal epithelium in *ex-vivo *culture showing complete wound closure within 24 h. The central green (fluorescein-stained) region in A and B shows the wound area (the white ring superimposed on the cornea in A-C is a reflection artefact that occurred during photography). D: X-gal staining of a healed *XLacZ*^+/- ^mosaic eye 24 h after wounding. E-H: Images from time-lapse confocal microscopy of healing of a 1 mm diameter central wound in a *PAX6-GFP *mosaic corneal epithelium (the white areas are GFP-positive) showing that the wound has closed by 18.75 h. after wounding. I-L: Healing of a peripheral wound showing cell migration and formation of a second whorling pattern. By 18.75 hours this wound has not closed completely.

Two approaches were used to study the dynamics of epithelial migration during *ex-vivo *wound healing more closely. First, the extent of cell mixing was monitored in *XLacZ*^+/- ^mosaic corneal epithelia following 24 hours wound healing. 1 mm diameter central wounds closed completely during this period (as demonstrated by absence of fluorescence when fluorescein was applied to the tissue [21]). X-gal staining revealed that striping patterns extended from the edge of the wound to its centre with little lateral mixing between stripes (Fig. 6A–D). This provides the first demonstration that striping patterns in X-inactivation mosaic eyes are restored as a central wound heals in whole eye-organ culture, indicating that corneal epithelial wounds heal in a clonal manner with no significant lateral mixing between stripes. For the second approach, which we term 'temporal mosaic analysis', time-lapse confocal microscopy was used to follow wound healing in mosaic corneas of Y001deltaDRR (*PAX6-GFP*) transgenic reporter mice [30]. Using this approach, radial GFP stripes and the wound margins were clearly visible in both central and peripheral wounds throughout the healing period without the use of fluorescein (Fig. 6).

Time-lapse recording of the radial GFP stripes allowed visualisation of the movement of GFP-positive cells towards the centre of wounds during the initial 24 h of the wound healing response. GFP-positive stripes extended towards the centre of the previously wounded region and stripes remained contiguous throughout the wound healing response (Fig. 6; for videos see additional files 1 and 2). While central wounds healed by centripetal movement of cells, healing of peripheral wounds involved both centripetal and centrifugal cell movement (relative to the centre of the cornea). This demonstration that cells can move centrifugally (towards the limbus) during wound-healing shows that population pressure from centripetally streaming cells produced by LSCs at the periphery of the cornea is not the main driving force. During healing of central wounds it appears that the original stripe pattern is effectively restored, as cells move inwards from the wound edge to meet close to the original point of convergence, although there could be minor differences. In contrast, during healing of peripheral wounds the stripes form a second point of convergence so that the new pattern of stripes is quite different from the original (Fig. 6H,L).

### Wounds in mosaic corneal epithelia sometimes heal asymmetrically

**Figure 7:**
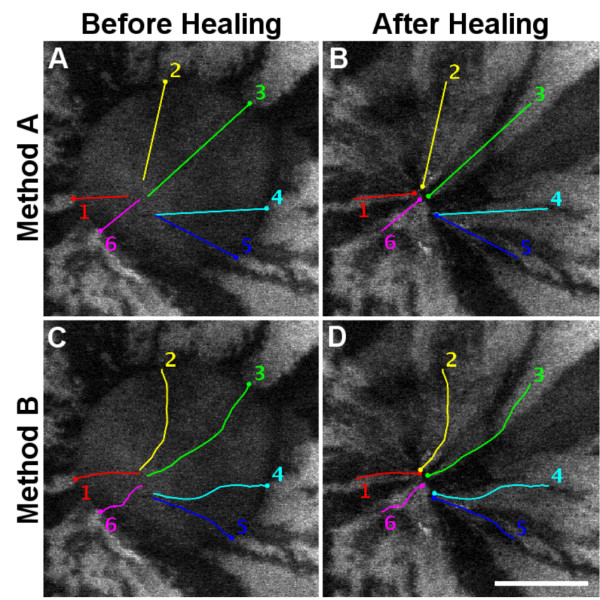
**Tracking of cell movement by monitoring stripe positions during the closure of a central wound**. Graphical representation of the healing of a central wound in a *PAX6-GFP *cornea. A: Time zero showing the extent of the 1 mm wound and the direction of movement of the leading edge of the stripe during wound closure. B: After 18 hours of healing the wound is completely closed but the stripes do not meet in the centre of the original wound. C: Time zero showing the routes taken by tracked stripes. D: After healing it is clear that stripes do not all extend equally, so cells in different places around the wound move at different rates and the wound closes asymmetrically. Scale bar in D = 500 μm.

The mean rate of migration calculated from the time taken for the wound shown in Fig 6E–H to completely heal was 0.44 μm min^-1^. However, tracking of individual stripes revealed that the wound actually closed asymmetrically (Fig. 7). Because image quality was relatively low and it was difficult to track some stripes, the total distance moved was calculated as the overall distance measured in a straight line between the first and last time points and as the aggregate of individual measurements between each time point (methods A and B respectively, in Table 1). This demonstrated that stripes from different places around the wound extended at different rates and is confirmed by the variation in the mean rates of movement for the leading edge of each of the six stripes (Table 1). Using method A to measure the distance moved, the rates of movement varied from 0.29 μm min^-1 ^(stripe 6) to 0.80 μm min^-1 ^(stripe 3) for the 6 different stripes measured. Method B gave very similar results (0.31 μm min^-1 ^for stripe 6 and 0.82 μm min^-1 ^for stripe 3). To our knowledge this is the first report of differential rates of cell movement at the leading edge of a circular wound in the corneal epithelium and highlights the advantages of using a mosaic analysis.

**Table 1:** Mean distance moved and rate of movement for 6 individual stripes during healing of wound shown in Fig. 6

**Stripe**	**Method A**^†^		**Method B**^††^	
		
	**Total distance** **(μm)**	**Mean rate** **(μm min^-1^)**	**Total distance** **(μm)**	**Mean rate** **(μm min^-1^)**
1	301	0.33	338	0.38
2	562	0.62	594	0.66
3	718	0.80	738	0.82
4	578	0.64	612	0.68
5	472	0.52	487	0.54
6	258	0.29	283	0.31

^† ^In method A the distance each stripe moved was calculated from the total distance moved regardless of the route.

^†† ^In method B the distance each stripe moved during wound healing was calculated by measuring the distance moved between every time point.

### Quantitative clonal analysis implies a reduction in the number of active LSC clones with age

**Figure 8:**
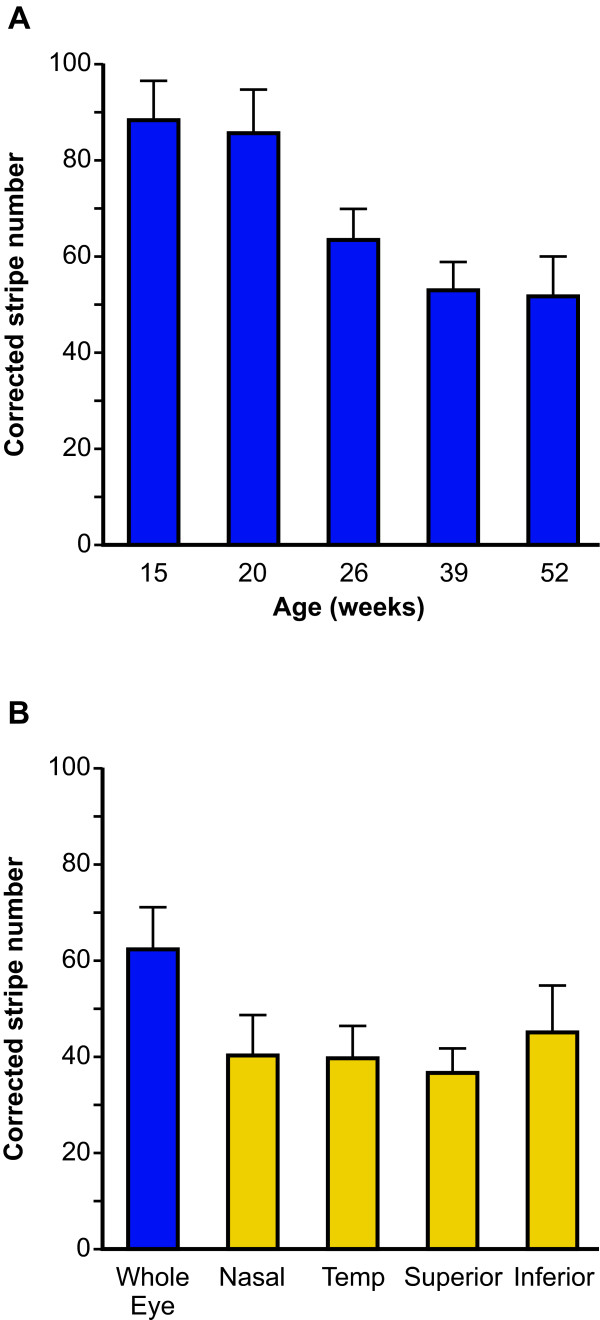
**Quantitative analysis of stripe patterns in corneal epithelium**. A: Mean corrected stripe numbers at 5 ages. A significant reduction is evident between 15 and 39 weeks of age from 88.33 ± 8.06 to 52.71 ± 5.79 (ANOVA *P *< 0.0001, Fisher's PLSD *P *< 0.0001) with no further reduction between 39 and 52 weeks of age (Fisher's PLSD *P *= 0.8224). Error bars = 95% CI. B: Mean corrected stripe numbers did not differ significantly between nasal and temporal or between superior and inferior regions (ANOVA, *P *= 0.5392); as expected, all four regions differed from the whole-eye (ANOVA *P *= 0.0002; Fisher's PLSD *P *< 0.05 in all cases). Temp = temporal.

Corrected stripe numbers were calculated for stripe patterns in 186 adult *XLacZ*^+/- ^mosaic corneal epithelia (see Methods) and used to compare LSC function at different ages up to 52 weeks and in different regions around the circumference. This showed that the corrected stripe number declined with age, up to but not beyond 39 weeks (Fig. 8A; Additional file 3). There was, however, no significant age-related change in the percentage of β-gal-positive cells in the corneal epithelium (ANOVA *P *= 0.8194; Additional file 3), reflecting the fact that X-inactivation is a single stable event in the early embryo. The decline in corrected stripe number implies there is an age-related decline in the number of active LSC clones and, therefore, an age-related decline in LSC function during this period.

### Distribution of active LSC clones around the circumference

Several studies based on analysis of holoclones in humans [33] and BrdU label-retaining cells in mice [34,35] suggest that limbal stem cells may be unevenly distributed around the limbal circumference. There is similar evidence that the proposed TAC marker α9β1 integrin is unevenly expressed around the circumference of the corneal epithelium [36]. Therefore, we compared the distribution of corrected stripes in two pairs of overlapping regions, each representing half of the circumference (nasal versus temporal and superior versus inferior) of a subset of *XLacZ*^+/- ^mosaic eyes. This showed that there was no significant difference among the four regions in either percentage of β-gal-positive cells (ANOVA, *P *= 0.7270) or the corrected stripe numbers (ANOVA, *P *= 0.5392; Fig. 8B; Additional file 4). Thus, our analysis showed no evidence for an uneven distribution of LSC clones around the limbal circumference.

## Discussion

This study has shown that once the LSCs are activated, coherent streams of clonally related cells move centripetally from the limbus, with little lateral mixing, to maintain the corneal epithelium. During corneal wound healing similar coherent streams of cells move into the wound from the perimeter, again with little lateral mixing. However, the direction of movement is not always away from the limbus and cells at different places around the wound may move at different rates, causing asymmetrical healing. The mosaic patterns, produced by streams of cells migrating from the limbus during normal corneal maintenance, show both stochastic and age-related variation. Importantly, analysis of these age-related changes implies that LSC function declines with age.

### Cell streaming during maintenance and repair of the corneal epithelium

Normal corneal epithelial homeostasis has been described in terms of an XYZ hypothesis [37]. This involves production of corneal epithelial cells (X) in the form of TACs from LSCs, centripetal movement of TACs (Y) and differentiation and eventual loss of terminally differentiated cells (Z) by desquamation, such that X+Y balances Z. The emergence of stripes in the mosaic corneal epithelium at around 3 weeks requires that stem cells are already specified and activated so the time that stripes first appear provides a guide to the time of stem cell activation. The time of emergence and subsequent maturation of stripes coincides with changes in the distribution of some markers associated with LSCs and TACs between 1 and 8 weeks after birth [36,38,39], which may indicate the maturation of a LSC niche in the basal limbal epithelium.

The streams of TACs leaving the limbus produce radial stripes that meet near the centre of the cornea and often form a clockwise or anticlockwise whorl. The whorls may reflect failure of centripetally migrating cells to meet precisely at the centre of the tissue or may be generated by a rotational force or chemotactic response, as described in other systems [40]. Age-related differences in the frequencies of striped patterns suggest that differences between midline, clockwise and anti-clockwise patterns become clearer after 20 weeks. This is also when the decline in LSC function occurred but it is unclear whether these two observations are related. It is possible that the whorls become tighter and more clearly delineated with age but this has not yet been investigated. Similarly, it is not yet known whether the whorling patterns are stable or dynamic, perhaps allowing stripes to change over time between clockwise and anticlockwise via mid-line or other interim patterns. The lack of association between the type of whorling pattern in left and right eyes from the same mouse suggests that the stripe patterns are produced stochastically in individual eyes. The stripes are produced by proliferation of β-gal positive and negative limbal stem cell clones, distributed around the corneal periphery, and centripetal movement of streams of cells but the differences will mostly be driven by unknown factors that cause the whorls.

Movement of corneal epithelial cells is more rapid during the early stages of wound healing than during normal corneal epithelial maintenance. Migration rates in the range of 0.43–1.06 μm min^-1 ^in response to wound healing in-vivo, ex-vivo and in-vitro have been reported [20,21,41,42] and similar rapid rates of movement occurred during healing of our *XLacZ*^+/- ^and GFP mosaic corneas. This is approximately 40-fold faster than the estimates of 17–26 μm day^-1 ^[9,43] for cell movement in the unwounded mouse corneal epithelium during normal corneal maintenance. Nevertheless, the rapid movement induced by removal of spatial constraints did not promote lateral cell mixing in the mosaic corneas and stripes remained contiguous despite the requirement for changes in cell-cell adhesion and remodelling of adhesion complexes necessary to close a wound (reviewed in [16]).

The fact that both *XLacZ*^+/- ^and *PAX6-GFP *stripes remained contiguous during healing shows that wounds healed in a clonal manner, suggesting a coordinated tissue response to wound healing stimuli, without any significant cell mixing. This differs from interpretations of experiments on adult skin epithelial wound healing, suggesting quite extensive cell mixing [24] and may reflect differences in the mechanism of wound closure. Adult skin wounds heal by lamellipodial crawling whereas wound healing in the adult corneal epithelium may use lamellipodial crawling and/or a mechanism involving a 'purse string' of contractile actin cable linking cells at the leading edge and co-ordinating cell movement [24,44]. However, it has been reported that the actin cable does not form during corneal wound healing in organ cultures [44] so this 'purse string' mechanism may not explain why little or no cell mixing occurred during our *XLacZ*^+/- ^and *PAX6-GFP *corneal wound healing experiments.

The observation that during the healing of peripheral wounds cells can move centrifugally (towards the limbus) shows that the main driving force is not population pressure from cells produced by LSCs so is likely to be population pressure from the wound-margin or other forces, such as chemotaxis or electric fields. Peripheral wound healing in mosaic corneas produced a new pattern of stripes, which converged within the wound. As the original central region was not wounded the healing of peripheral wounds produced a second centre of stripe convergence. The resultant bi-convergent striping pattern resembles the abnormal striping patterns seen previously in some *XLacZ*^+/- ^mosaic eyes (Fig. 2J and data not shown), suggesting that they may have been produced by previous wound healing events.

Use of time-lapse confocal microscopy for studies of wound healing allows rates of cell movement to be monitored throughout wound healing and provides a simple means of identifying the time of wound closure. (Without constant monitoring, the rate of wound closure can only be determined if the experiment is terminated before the wound closes, so that changes in wound size can be estimated during a period while the wound is still healing [21].) The combination of time-lapse microscopy and mosaic analysis offers the additional advantage of being able to measure the rate of cell movement in different regions around the wound circumference. The observation that wounds may heal asymmetrically hints at mechanisms involved in inducing cell movement. It suggests that cells are unlikely to be pushed with equal force from all around the wound edge, so either the population pressure is unequal or cells are attracted to a specific region that need not coincide with the centre of the wound. For example, if the wound was not perfectly central and the converging streams of cells met at the centre of the cornea (e.g. if the central stroma acted as a signalling centre) the migrating cells would probably not meet at the centre of the wound. The experimental system described here clearly offers opportunities to design further experiments to distinguish between alternative mechanisms that control the direction of corneal epithelial cell movement during normal maintenance and wound healing.

### Distribution of limbal stem cell clones

Although previous reports suggest there is an uneven distribution of LSCs and TACs around the circumference, no clear consensus has emerged. Analysis of holoclones produced by human limbal cells suggested there were more colony forming cells (putative LSCs) in the superior and temporal than the inferior and nasal regions [33] but this was based on a single eye. Uneven distributions of BrdU label-retaining cells (which include putative LSCs) in mice have been reported to be higher in the superior and inferior compared to nasal and temporal quadrants [34] or higher in superior and temporal than inferior and nasal regions [35]. In contrast, more cells expressing α9β1 integrin (proposed TAC marker) were reported in the nasal region of the corneal epithelium compared to temporal, superior, and inferior regions [36]. Our analysis did not provide any evidence for an uneven distribution of LSC clone numbers but this does not relate directly to the numbers of LSCs. Also, to reduce edge-effects, our analysis compared LSC clone numbers between two halves rather than four quadrants so would not detect all uneven distributions. For example, if there were more LSC clones in the superior and inferior than temporal and nasal regions they would be equally represented when comparing superior versus inferior and temporal versus nasal.

### Reduction of limbal stem cell function with age

**Figure 9:**
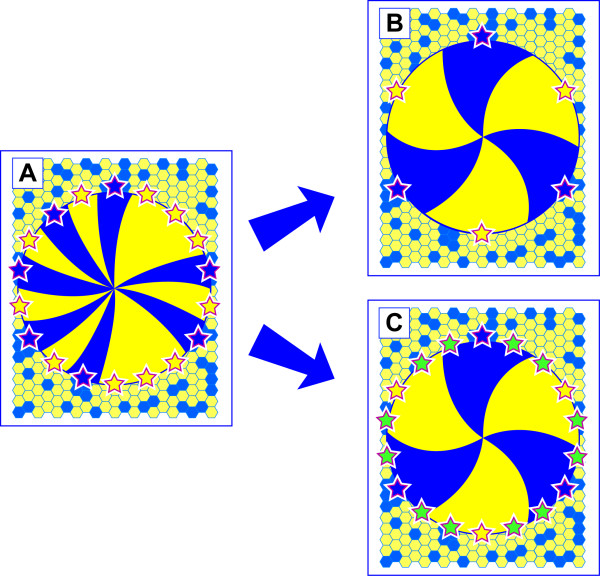
**Possible mechanisms for age-related decline in limbal stem cell function**. Diagram showing two possible ways in which limbal stem cell function may decline with age. A: Full complement of active limbal stem cells (shown as blue and yellow stars) in young eye. B: Reduction in number of LSCs in older eye. C: Increase in proportion of quiescent LSCs in older eye. The mosaic corneal epithelium is shown as a disk with radial blue and yellow stripes produced by LSCs, represented by stars, at the edge of the cornea. Blue and yellow stars represent active LSCs; green stars represent quiescent LSCs.

Our quantitative analysis implies that LSC function declines with age to 39 weeks and then stabilises. An age-related decline could occur either if some LSCs were lost (e.g. by symmetrical division to produce two TACs instead of 1 LSC plus 1 TAC) or if some LSCs become quiescent, resulting in a reduction in the number of active LSC clones (Fig. 9). Further work with suitable LSC markers, once they become available, is needed to determine whether there is a parallel decline in LSC numbers, similar to the observed decline in epidermal holoclones [45]. Alternatively, the influence of ageing might be less direct (e.g. primarily via the stem cell niche or a systemic effect resulting in quiescence or dysfunction of some of the stem cell clones), as seen in some other stem cell systems (reviewed in [12]). Although the LSC function declines with age most stripes span the corneal radius at all ages suggesting that corneal epithelial cell migration is normal and sufficient cells are produced to maintain corneal homeostasis up to 52 weeks of age. Furthermore, our evidence for an age-related decline in LSC function prompts additional experimental questions concerning age-related changes in the limbal niche, LSC numbers, the balance between quiescent and active LSC states and the minimum number of active LSCs required to maintain the corneal epithelium.

## Conclusion

Adult stem cell therapy, the holy grail of stem cell research, is already advanced for the treatment of some corneal conditions [46]. However, understanding the cellular mechanisms underpinning these surgical interventions requires a better understanding of LSC function and corneal maintenance from well-characterised animal models as well as human tissues. In particular, tissue ageing and its implications for adult stem cells and their niches needs further study. Here, analysis of age-related changes implies that LSC function declines with age. Together with evidence from other systems, linking stem cell deterioration to the ageing process [13], this is likely to encourage investigations of how LSCs and the limbal niche change with age.

## Methods

### Animals

H253 strain mice [1], ubiquitously expressing an X-linked *nLacZ *transgene (abbreviated to *XLacZ*), were obtained from the MRC Mammalian Genetics Unit, Harwell, UK. Hemizygous *XLacZ*^+/- ^females (produced from crosses between *XLacZ*^+/*Y*^, H253 males and (C57BL/6 × CBA/Ca)F1 females) show mosaic transgene expression, after X-gal staining for β-gal reporter activity, due to random X-inactivation during development. Mice of the transgenic reporter lines Y001deltaDRR and Y223 (showing mosaic expression of *PAX6-GFP*) were used to visualise stripe dynamics by time-lapse confocal microscopy. The Y223 line carries 6 copies of a yeast artificial chromosome (YAC) containing the human *PAX6 *locus [31] in which PAX6 protein expression is prevented by a GFP reporter gene inserted into the PAX6 ATG start codon. In addition, the downstream regulatory region (DRR) of the PAX6 locus is flanked by loxP sites. Line Y001deltaDRR was produced by crossing Y001 (containing a single copy of the above YAC with a 10–20 kb truncation) with the *CAGGS-Cre *line [32] resulting in germline deletion of the DRR region and leading to reduced GFP expression in the lens [30].

### Histology

Whole eyes dissected at 3 to 52 weeks after birth were fixed and stained for β-gal reporter activity as described previously [4]. To prepare whole-mounts of the ocular surface, the eye was first enucleated with the eyelid intact, the posterior segment and lens were then removed and several radial cuts were made in the cornea and conjunctiva to allow the tissue to be flattened. Where sections were required the lens was removed before wax embedding. 7 μm paraffin sections were cut on a microtome and mounted onto standard microscope slides. The sections were first dewaxed in Histoclear (2 × 5 mins) and then dipped in 95% (vol/vol) ethanol (1 min). Sections were then counterstained by dipping in Eosin (Surgipath) for 1 min, washing in tap water (5 mins) and staining in 1% (wt/vol) Neutral Red solution (3 mins). Slides were then rinsed in distilled water (2 × 1 min), dehydrated by dipping in a graded ethanol series (70%, 95% and 100% vol/vol) followed by xylene (2 × 1 min). Cover slips were mounted with DPX mounting medium (VWR, Poole, UK).

### Wound healing and time-lapse microscopy

As described previously 1 mm diameter wounds through the full epithelial thickness were made in *XLacZ*^+/- ^corneas. Eyes were then cultured for 24 hours and stained with fluorescein at 0, 6 and 24 h to visualise and photograph the wound margin [21]. Eyes were returned to culture after the examination at 6 h and after 24 h they were fixed and stained with X-gal as described previously [4]. For preliminary evaluation of different transgenic lines, GFP expression in the corneas was imaged using a Leica MZFLIII fluorescence stereo microscope fitted with a Coolsnap cf colour CCD camera (Photometrics Ltd, Tucson, AZ). Eyes from line Y100deltaDRR were wounded and enucleated in the same way as *XLacZ*^+/- ^eyes. They were then cultured in a controlled environmental chamber (37°C; 5% CO_2 _in air) surrounding the stage of a Leica inverted confocal microscope. During culture the eye was supported on a specially designed stand so the cornea faced downwards. Z-stack images were collected at intervals over a 24 h period using a ×5 objective. Confocal images were analysed using ImageJ, a shareware image analysis software package [47,48]. The 'Stackreg' plug-in [49] developed by Philippe Thévenaz, (Biomedical Imaging Group, Swiss Federal Institute of Technology Lausanne, Switzerland) was used to correct for any movement of the eye between time points. The distance moved by the leading edges of individual stripes were measured in two ways. For method A, the distance moved in a straight line was simply measured between the first and last time points in the experiment. For method B, stripes were analysed by manually tracking and measuring the distance moved between time points. Both methods used the 'MtrackJ' plug-in developed by Erik Meijering (Biomedical Imaging Group Rotterdam, Erasmus MC – University Medical Center Rotterdam, The Netherlands).

### Quantitative analysis of mosaic patterns

For analysis of variegated, mosaic patterns, it is important to distinguish between different types of patches and clones. We use three terms defined previously [25,50-53]. A *patch *is a group of cells of like genotype (or phenotype) that are contiguous at the time of consideration. A *descendent clone *is any group of clonally related cells irrespective of whether they have remained contiguous throughout development. A *coherent clone *is a group of clonally-related cells that have remained contiguous throughout development. The number of coherent clones per patch depends partly on the proportions of the two cell populations in the mosaic. For one-dimensional mosaics (strings of two cell populations) the number of coherent clones per patch of cell population 'A' is estimated as 1/(1-*p*) (where *p *is the proportion of cell population 'A' in the mosaic) [25,28,53].

The observed mean width of β-gal-positive stripes in the corneal epithelium was corrected for the probability that stripes would contain multiple adjacent β-gal-positive corneal epithelial clones. This involved dividing the observed mean width by the function 1/(1-*p*), where *p *is the proportion of β-gal-positive cells around the circumference [4,25,52,53]. For radial stripes that form a complete circle, the corrected mean stripe width is identical for β-gal-positive and β-gal-negative stripes because the numbers of β-gal-positive and β-gal-negative stripes are identical and the proportions of β-gal-positive and β-gal-negative cells around the circumference are not independent. The reciprocal of this corrected mean stripe width, expressed as the proportion of the circumference, is the corrected stripe number. This provides an estimate of the total number of corneal epithelial coherent clones (both β-gal-positive and β-gal-negative) per circumference [4,5]. This estimate assumes (1) coherent clones are equal in size, or their sizes are normally distributed, and (2) β-gal-positive and β-gal-negative coherent clones are randomly distributed around the circumference. Even if these assumptions are not correct the corrected stripe number should be proportional to the number of coherent clones so its application as a comparative measure is still valid.

### Clonal analysis

The clonal analysis of striping patterns between 15 and 52 weeks was carried out as described previously [4,5]. To determine whether stripe patterns varied around the circumference, mean corrected stripe numbers were estimated separately in four regions as well as the whole circumference for 15 mosaic eyes (from 23–24 week old *XLacZ*^+/- ^mice). Each region was equivalent to half of the circumference (nasal or temporal; superior or inferior). The nasal conjunctival epithelium was sutured to mark its orientation and eyes were removed and stained. Stripes were counted and measured separately in different hemispheres in two ways: nasal versus temporal and superior versus inferior. Stripes on the nasal-temporal or superior-inferior boundaries were included in both regions. To correct for overlapping stripes, the corrected stripe number per region was calculated by dividing half the circumference by the corrected mean stripe width separately for each of the four regions.

## Abbreviations

ANOVA: analysis of variance; β-gal: β-galactosidase; CI: confidence interval; GFP: green fluorescent protein; LSC: limbal stem cell; *PAX6-GFP*: Y001deltaDRR (*PAX6-GFP*) transgenic mice; TAC: transient amplifying cell (also known as transit amplifying cell); *XLacZ*^+/-^: female X-inactivation mosaic mice, hemizygous for the H253 X-linked *nLacZ *transgene.

## Competing interests

The authors declare that they have no competing interests.

## Authors' contributions

RM devised the temporal mosaic analysis of wound healing, carried out the quantitative clonal analysis and histology, contributed to the design of the study and drafted the manuscript. TR carried out the wound healing (in conjunction with RM). DK produced and provided the Y001deltaDRR mice. JW and SM conceived the study, participated in its design and coordination and helped to draft the manuscript. All authors read and approved the final manuscript.

## Supplementary Material

Additional file 1 **Time-lapse confocal microscopy of healing of a 1 mm diameter central wound in a *PAX6-GFP *mosaic corneal epithelium**. Time-lapse video showing the complete healing of a central wound in the corneal epithelium between 5.25 and 18.75 hours post wounding. At 18.75 hours the wound appears completely healed and stripes extend to the centre of the previously wounded region. Click here for file

Additional file 2 **Time-lapse confocal microscopy of healing of a 1 mm diameter peripheral wound in a *PAX6-GFP *mosaic corneal epithelium**. Time-lapse video showing incomplete healing of a peripheral wound in the corneal epithelium between 5.25 and 18.75 hours post wounding. At 18.75 hours the wound is only partially healed, stripes extend towards the centre of the previously wounded region. Click here for file

Additional file 3 **The effects of age on mean corrected stripe number in the corneal epithelium of X-inactivation mosaic mice**. Click here for file

Additional file 4 **Comparisons of corneal epithelial stripe numbers in different regions (at 23–24 weeks)**. Click here for file
