# Supplementary material for: Mosaic analysis of stem cell function and wound healing in the mouse corneal epithelium
Source: BMC Dev Biol. 2009 Jan 7;9:4. doi: 10.1186/1471-213X-9-4 (PMC2639382; doi:10.1186/1471-213X-9-4)
Supplement: Additional file 3 — The effects of age on mean corrected stripe number in the corneal epithelium of X-inactivation mosaic mice. [file 1471-213X-9-4-S3.pdf]

Additional File 3: The effects of age on mean corrected stripe number in the corneal epithelium of X-inactivation mosaic mice

A. Analysis of variance

Age (weeks)	Number of eyes	% ß-Gal positive ± 95% CI	Mean corrected stripe number ± 95% CI
15	36	72.7 ± 5.14	88.3 ± 8.06
20	38	70.1 ± 6.80	85.5 ± 9.18
26	38	70.1 ± 4.75	63.3 ± 6.41
39	40	67.6 ± 5.67	52.7 ± 5.79
52	34	70.3 ± 5.89	51.5 ± 8.29
**Statistical significance**			
1-way ANOVA		$P = 0.819$	$P < 0.0001$

B. Pairwise comparisons of mean corrected stripe number for different ages

Age (weeks)	20	26	39	52
**15**	$P = 0.611$	$P < 0.0001$	$P < 0.0001$	$P < 0.0001$
**20**		$P < 0.0001$	$P < 0.0001$	$P < 0.0001$
**26**			$P = 0.050$	$P = 0.036$
**39**				$P = 0.822$

P = Fishers PLSD probability.
