# Supplementary material for: Mosaic analysis of stem cell function and wound healing in the mouse corneal epithelium
Source: BMC Dev Biol. 2009 Jan 7;9:4. doi: 10.1186/1471-213X-9-4 (PMC2639382; doi:10.1186/1471-213X-9-4)
Supplement: Additional file 4 — Comparisons of corneal epithelial stripe numbers in different regions (at 23–24 weeks). [file 1471-213X-9-4-S4.pdf]

Additional File 4: Comparisons of corneal epithelial stripe numbers in different regions (at 23-24 weeks)

Corneal Region[†]	Number of eyes	% ß-Gal positive ± 95% CI	Mean corrected stripe number ± 95% CI
Whole cornea	15	68.0 ± 7.20	62.3 ± 8.77
Inferior	15	73.6 ± 9.73	35.8 ± 8.80
Superior	15	66.1 ± 8.83	30.4 ± 4.56
Nasal	15	70.4 ± 8.11	33.5 ± 6.36
Temporal	15	68.4 ± 10.91	33.7 ± 5.94
**Statistical significance**			
1-way ANOVA including whole cornea		P = 0.820	P = 0.0002
1-way ANOVA excluding whole cornea		P = 0.727	P = 0.539

[†] Each region represented half of the whole cornea.
